# Functional, Volumetric, and Growth Outcomes After Pediatric Bilateral Lung Transplantation

**DOI:** 10.1111/petr.70422

**Published:** 2026-08-04

**Authors:** Seon Yong Bae, Samina Park, Dae Hyeon Kim, Taeyoung Yun, Ji Hyeon Park, Bubse Na, Kwon Joong Na, Hyun Joo Lee, Ji Soo Park, Dong In Suh, Jimyung Park, In Kyu Park, Chang Hyun Kang, Young Tae Kim

**Affiliations:** ^1^ Department of Thoracic and Cardiovascular Surgery Seoul National University Hospital Seoul Republic of Korea; ^2^ Seoul National University Cancer Research Institute Seoul Republic of Korea; ^3^ Department of Thoracic and Cardiovascular Surgery Seoul National University College of Medicine Seoul Republic of Korea; ^4^ Department of Pediatrics Seoul National University College of Medicine Seoul Korea; ^5^ Division of Pulmonary and Critical Care Medicine, Department of Internal Medicine Seoul National University Hospital Seoul Republic of Korea

**Keywords:** lung transplantation, pediatric, pulmonary function, somatic growth

## Abstract

**Objective:**

Long‐term multidimensional evaluations of pediatric lung transplantation are limited. This study assessed the functional recovery, volumetric adaptation, and growth in pediatric recipients by comparing age and graft type.

**Methods:**

This single‐center, retrospective, descriptive study longitudinally reviewed 16 children (age < 18 years) who underwent bilateral deceased‐donor lung transplantation. Perioperative characteristics, pulmonary function, computed tomography (CT)–derived lung volume, somatic growth, and survival were analyzed. Overall survival (OS) and chronic lung allograft dysfunction (CLAD)–free survival were estimated using the Kaplan–Meier method, and longitudinal physiological and developmental patterns were assessed descriptively.

**Results:**

In the younger group (≤ 10 years, *n* = 7) and the older group (> 10 years, *n* = 9), lobar transplantation was performed in 5 (71.4%) and 3 (33.3%) patients, respectively. CLAD was diagnosed in 1 younger recipient (14.3%) and 2 older recipients (22.2%). OS did not differ significantly between age groups (log‐rank *p* = 0.96), and CLAD‐free survival was comparable (log‐rank *p* = 0.73). CT volumetry demonstrated progressive postoperative lung expansion with similar temporal trajectories across age groups and between lobar and whole‐lung grafts. Growth patterns demonstrated meaningful postoperative improvement, with many children who exhibited faltering pretransplant growth demonstrating catch‐up toward individualized percentile curves.

**Conclusions:**

Pediatric lung transplantation results in favorable long‐term recovery, including functional improvement, durable volumetric adaptation, and partial restoration of growth dynamics. Comparable outcomes between lobar and whole‐lung grafts support the use of size‐reduced grafts in cases of donor–recipient mismatch.

## Graphical abstract

Pediatric bilateral lung transplantation achieved durable functional recovery, catch‐up somatic growth, and comparable postoperative lung volumetric adaptation across graft types, supporting the feasibility of size‐reduced transplantation regardless of recipient age.

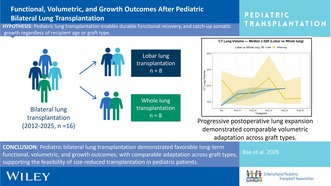

**Abbreviations**
CLAD: chronic lung allograft dysfunction
CPB: cardiopulmonary bypass
CT: computed tomography
DDLT: deceased‐donor lung transplantation
ECMO: extracorporeal membrane oxygenation
FEV_1_
: forced expiratory volume in 1 s
FVC: forced vital capacity
IQR: interquartile range
ISHLT: International Society for Heart and Lung Transplantation
OS: overall survival
PAH: pulmonary arterial hypertension
PFT: pulmonary function test
*Z*‐score: standard deviation score (age‐ and sex‐adjusted)

## 1 Introduction

Pediatric lung transplantation, although uncommon, remains an essential treatment for children with irreversible end‐stage lung disease. Despite improvements in outcomes, pediatric candidates continue to experience disproportionately high waitlist mortality and reduced long‐term survival owing to limited donor availability and the complexity of managing young recipients throughout the transplant process [1, 2, 3]. As suitably sized donor lungs are scarce, surgeons frequently employ size‐reduced or lobar grafts to accommodate the smaller thoracic cavities in children.

According to the International Society for Heart and Lung Transplantation (ISHLT) Registry, short‐term survival after pediatric lung transplantation has shown gradual improvement across eras, with more recent cohorts demonstrating higher 1‐year survival trends than earlier periods [2, 4]. However, overall survival (OS) remains substantially lower than that involving other solid organs, largely because of the persistent impact of chronic lung allograft dysfunction (CLAD) [2]. Prior registry analyses consistently show a marked decline in survival beyond the early postoperative period, with CLAD being the predominant cause of late graft failure and reduced 5–10‐year survival [2, 4]. These findings highlight the ongoing challenges in pediatric lung transplantation, including donor scarcity and long‐term vulnerability of allografts in growing children.

These limitations are further exacerbated by the unique physiological and developmental characteristics of pediatric recipients. Unlike adults, pediatric recipients undergo continuous somatic and respiratory development, which requires transplanted lungs to adapt structurally and functionally over time. However, comparative data evaluating the performance of lobar versus whole‐lung grafts in this context remain sparse. Although several single‐center or retrospective series have reported outcomes after living‐donor lobar transplantation, direct head‐to‐head comparisons with whole‐lung grafts are still limited [5, 6].

Pediatric transplantation also intersects with key developmental processes such as chest wall maturation and catch‐up growth following chronic illness, which may influence graft performance. Prior studies offer mixed findings, with immature donor lungs in infants exhibiting near‐physiological growth [7], whereas adult lobar grafts may demonstrate restricted alveolar expansion and reduced diffusing capacity compared to whole‐lung grafts [5, 6]. However, systematic evaluations of pre‐ and post‐transplant somatic growth patterns in pediatric recipients remain limited.

Given these gaps, longitudinal data integrating physiological, radiological, and developmental outcomes are needed to clarify recovery trajectories and identify factors influencing long‐term success. Accordingly, we aimed to characterize the perioperative outcomes, functional recovery, volumetric adaptation, growth patterns, and survival in pediatric lung transplant recipients.

## 2 Materials and Methods

### 2.1 Study Design

This retrospective cohort study included 16 pediatric patients (≤ 18 years) who underwent bilateral lung transplantation at Seoul National University Hospital between January 2012 and July 2025. Clinical follow‐up data are available through September 2025. This study was approved by the Institutional Review Board (IRB) of Seoul National University Hospital (IRB No. 2509‐081‐1675), and the requirement for informed consent was waived because of its retrospective design.

### 2.2 Indications and Operation Procedure

All patients in this cohort underwent deceased‐donor lung transplantation (DDLT). Donor and recipient eligibility were determined according to the regulations of the national organ‐sharing network. The operative techniques and postoperative management used in the DDLT cohort were consistent with those previously described [7, 8].

Intraoperative support with extracorporeal membrane oxygenation (ECMO) or cardiopulmonary bypass (CPB) was implemented based on patient status and vascular access feasibility, and vessel calibers (femoral artery/vein and internal jugular vein/artery) were assessed preoperatively. Due to the young age and compliance constraints inherent to the pediatric population, active or awake ECMO was not implemented in this cohort, and postoperative mechanical support was maintained under appropriate sedation to prevent cannula‐related complications.

Decisions regarding whole‐lung versus lobar graft transplantation were guided primarily by donor–recipient size mismatch. Size matching followed established pediatric lung transplant criteria based on predicted lung volume, chest cavity dimensions, and published surgical sizing principles [6, 9]. When a significant disparity was noted, graft volume reduction (via wedge resection or lobectomy) was considered, depending on the condition and dimensions of the donor lung lobes. This active volume‐reduction strategy represents a major programmatic policy at our institution, employed to maximize transplant opportunities under severe local donor shortages. Postoperative care protocols mirrored those previously reported for pediatric lung transplantation [3, 10].

### 2.3 Pulmonary Function Test (PFT), Computed Tomography (CT), and Volumetric Analysis

PFTs were performed at routine intervals, generally every 6–12 months, after transplantation. Because reliable spirometry requires adequate cooperation, PFTs were obtained only in children 7 years of age or older. Measurements included forced expiratory volume in 1 s (FEV_1_) and forced vital capacity (FVC), and were recorded longitudinally.

Chest CT was also performed at regular 6‐ to 12‐month intervals. Quantitative volumetric assessment was conducted using a three‐dimensional imaging platform (SYNAPSE VINCENT; Fujifilm Co. Ltd., Tokyo, Japan). The software generated three‐dimensional reconstructions of the tracheobronchial tree and lung parenchyma and automatically calculated the lobar and total lung volumes. Serial CT‐based volumetry was used to evaluate postoperative changes in graft size over time [11].

### 2.4 Definition of CLAD and Somatic Growth Patterns

CLAD was defined according to the ISHLT consensus criteria as a sustained ≥ 20% decline in FEV_1_ from the patient's best post‐transplant baseline for at least 3 months after excluding reversible causes such as infection, acute rejection, or airway complications [12].

Somatic growth trajectories were derived from height and weight measurements obtained during routine outpatient visits before and after transplantation. Growth outcomes were analyzed longitudinally using age‐ and sex‐adjusted *Z*‐scores.

### 2.5 Statistical Analysis

All statistical analyses were performed using IBM SPSS Statistics (v.25.0; IBM Corp., Armonk, NY, USA) and R software (R Studio, PBC, Boston, MA, USA). Continuous variables were summarized as means ± SD, and categorical variables were reported as counts and percentages.

Patients were stratified according to age group (≤ 10 vs. > 10 years) and graft type (lobar vs. whole‐lung transplantation). Between‐group comparisons were performed for perioperative characteristics, longitudinal pulmonary function, volumetric changes on CT, somatic growth indices, and survival outcomes.

OS was defined as the time from surgery to death from any cause or the date of the last available follow‐up. CLAD–free survival was defined as the time from transplantation to the first diagnosis of CLAD, death, or the last available follow‐up, whichever occurred first. OS and CLAD‐free survival were estimated using the Kaplan–Meier method and compared using the log‐rank test. The one recent patient with a follow‐up duration under 3 months was censored entirely from these time‐to‐event Kaplan–Meier estimates to prevent early follow‐up bias. Longitudinal trends in pulmonary function, CT‐derived lung volume, and growth indices were analyzed descriptively and graphically.

For growth‐related analyses, including height and body weight trajectories, normality was assessed using the Shapiro–Wilk test. Patients with incomplete or unreliable anthropometric records, or those with excessively prolonged and irregular outpatient follow‐up intervals, were further excluded from the descriptive trend lines. Comparisons between groups were conducted using Student's *t*‐test for normally distributed variables or appropriate nonparametric tests when normality assumptions were not met. Statistical significance was defined as a two‐sided *p*‐value < 0.05.

## 3 Results

### 3.1 Baseline Characteristics of Study Population

Study population baseline characteristics are summarized in Table 1. Sixteen recipients underwent bilateral DDLT, including seven younger recipients (≤ 10 years) and nine older recipients (> 10 years). As expected, age‐related anthropometric differences were evident: younger recipients had a median age of 6.0 years (interquartile range (IQR), 2.0–10.0) compared with 13.0 years (IQR, 11.0–17.5) in older recipients.

**TABLE 1:** Baseline, donor, and surgical characteristics of pediatric lung transplant recipients.

Variable	≤ 10 years(*n* = 7)	> 10 years (*n* = 9)
Recipient characteristics		
Age, years	6.0 [2.0–10.0]	13.0 [11.0–17.5]
Male sex, *n* (%)	5 (71.4)	5 (55.6)
Height, cm	111 [82–128]	148 [137.3–160.7]
Body weight, kg	16.25 [10.0–21.3]	42.0 [22.0–46.3]
Primary diagnosis		
Graft‐versus‐host disease	1 (14.3)	5 (55.6)
Pulmonary arterial hypertension	2 (28.6)	3 (33.3)
Cystic fibrosis	1 (14.3)	1 (11.1)
Other lung disease	3 (42.9)	—
Donor characteristics		
Donor age, years	19.0 [4.0–30.0]	33.0 [23.0–46.5]
Donor height, cm	155 [100–177]	160 [154.5–169.0]
Donor body weight, kg	50.2 [18.5–58.0]	50.0 [43.2–56.95]
Donor male sex, *n* (%)	4 (57.1)	2 (22.2)
Surgical characteristics		
Volume reduction, *n* (%)	5 (71.4)	3 (33.3)
Right lung		
Lobectomy	4 (57.1)	2 (22.2)
Segmentectomy	0 (0.0)	0 (0.0)
Wedge resection	1 (14.3)	1 (11.1)
Left lung		
Lobectomy	3 (42.9)	1 (11.1)
Segmentectomy	1 (14.3)	1 (11.1)
Wedge resection	0 (0.0)	1 (11.1)

*Note:* Continuous variables are expressed as median [interquartile range] as appropriate, and categorical variables as *n* (%).

The primary diagnoses differed across age groups. Graft‐versus‐host diseaseand pulmonary arterial hypertension (PAH) predominated among older recipients (5 (55.6%) and 3 (33.3%), respectively), whereas younger recipients exhibited a more heterogeneous mix of etiologies, including interstitial lung disease, acute respiratory distress syndrome, and bronchiectasis grouped as “other lung diseases.”

Donor characteristics exhibited modest variations. Donor age was lower for the ≤ 10‐year group (median 19.0 vs. 33.0 years old), while donor height and body weight were comparable. Male donors accounted for 57.1% of donors in younger recipients and 22.2% in the older cohort. Consistent with the anticipated chest cavity size mismatch, volume reduction procedures were required more frequently in younger recipients (5 (71.4%) vs. 3 (33.3%)).

### 3.2 Perioperative Outcomes

Pre‐ and intraoperative outcomes are summarized in Table S1. The median waiting time was shorter in the ≤ 10‐year group compared with the older cohort (37 [1–68] vs. 58 [31.5–186] days). Preoperative ECMO was performed at a similar rate (3 (42.9%) vs. 4 (44.4%)). The surgical approaches were comparable, with clamshell incisions in most recipients; only one 10‐year‐old recipient underwent anterior thoracotomy. CPB use (2 (28.6%) vs. 3 (33.3%)) and intraoperative ECMO use (5 (71.4%) vs. 6 (66.7%)) were similar. The operative parameters, including cold and warm ischemic times, did not differ significantly between the groups.

Patient postoperative outcomes showed comparable ECMO requirements (3 (42.9%) vs. 4 (44.4%)) (Table S2). All postoperative ECMO in the ≤ 10‐year group was Veno‐venous ECMO, including one case supported with an Avalon dual‐lumen cannula. Older recipients had longer intensive care unit stays (21 [13.5–30] vs. 9 [8–44] days) and longer total hospitalization (62 [39.25–190.5] vs. 43.5 [27.8–88.5] days). Airway anastomoses were intact in all evaluated recipients. Reoperation for bleeding occurred at similar rates (1 (14.3%) vs. 1 (11.1%)).

One recipient in each age group died before discharge (1 (14.3%) vs. 1 (11.1%)). Thirty‐day mortality due to intracranial hemorrhage occurred in one younger recipient. Neurological complications were more common in younger recipients (5 (71.4%) vs. 2 (22.2%)), with the younger group experiencing delirium (*n* = 1), seizures (*n* = 3), and intracranial hemorrhage (*n* = 1), whereas older recipients presented with delirium. Acute rejection did not occur in either group.

### 3.3 Long‐Term and Survival Outcomes

Immunosuppressive therapy consisted predominantly of tacrolimus in both age groups and was employed in 6 of 7 younger recipients (85.7%) and all older recipients (100%). One younger recipient ≤ 10 years received cyclosporine. CLAD was diagnosed in one younger recipient (14.3%) and in two older recipients (22.2%). The median time to CLAD onset was 84 months in the younger recipients, whereas the two older recipients developed CLAD at 32 and 115 months, respectively.

At the last follow‐up, 5 of 7 younger recipients (71.4%) and 6 of 9 older recipients (66.7%) were alive. Median survival duration was 46.1 months (IQR, 12.45–72.1) in the ≤ 10‐year group and 38.8 months (IQR, 17.6–71.9) in the > 10‐year group, excluding one recipient transplanted within 1 month of data lock. The causes of death differed by age; both deaths in the ≤ 10‐year group were due to intracranial hemorrhage, whereas deaths in the older group resulted from pneumonia (*n* = 2) and progression of underlying leukemia (*n* = 1).

**FIGURE 1:**
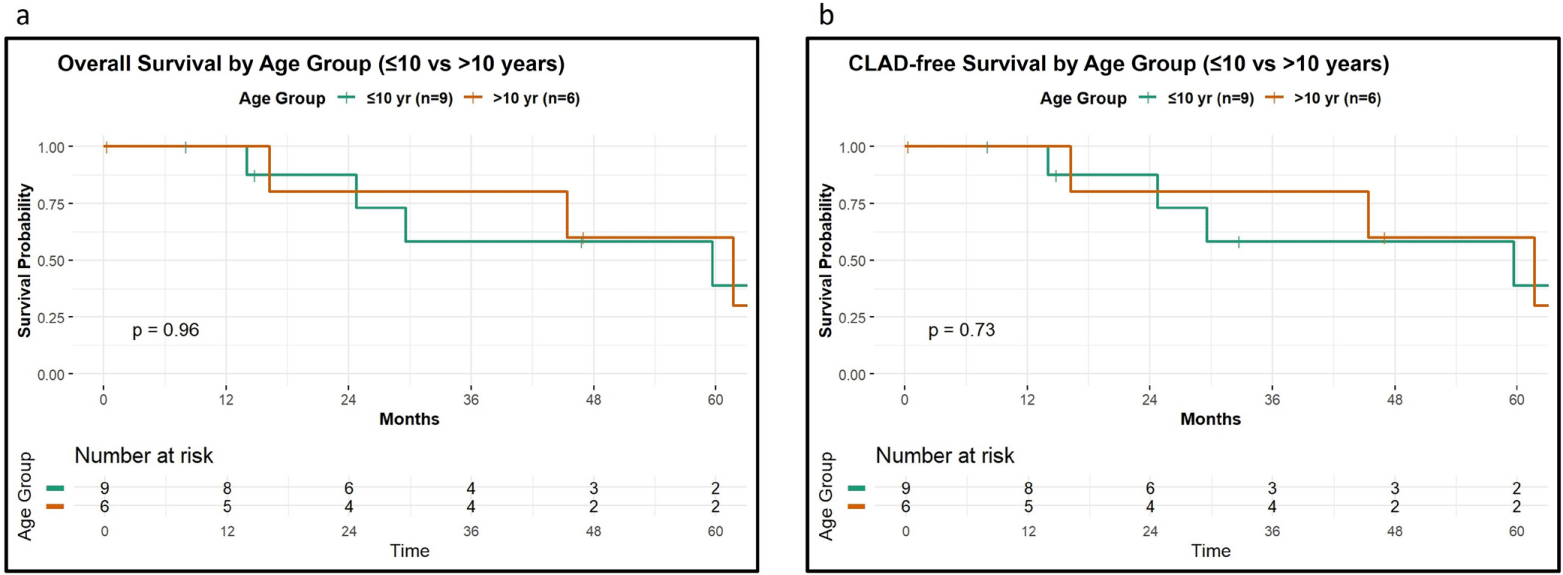
Five‐year overall survival and CLAD‐free survival according to age group Kaplan–Meier curves comparing outcomes between younger recipients (≤ 10 years; *n* = 9) and older recipients (> 10 years; *n* = 6). (a) Overall survival showed no significant difference between groups (log‐rank *p* = 0.96). (b) CLAD‐free survival was comparable (log‐rank test, *p* = 0.73). One recipient was excluded from survival analysis due to insufficient follow‐up (< 30 days). Although both curves demonstrated a decline beginning approximately 4–5 years after transplantation, their trajectories remained parallel, without evidence of age‐related divergence. Number‐at‐risk tables for each time point are presented below each panel. CLAD, chronic lung allograft dysfunction.

Five‐year OS and CLAD‐free survival rates did not differ significantly between age groups (Figure 1). Kaplan–Meier analysis showed nearly overlapping survival curves for younger and older recipients, with log‐rank *p*‐values of 0.96 for OS and 0.73 for CLAD‐free survival. Although both groups exhibited a decline at approximately 4–5 years post‐transplantation, the patterns remained parallel, without evidence of age‐related divergence.

### 3.4 Functional Recovery: Pulmonary Function and Lung Volume

**FIGURE 2:**
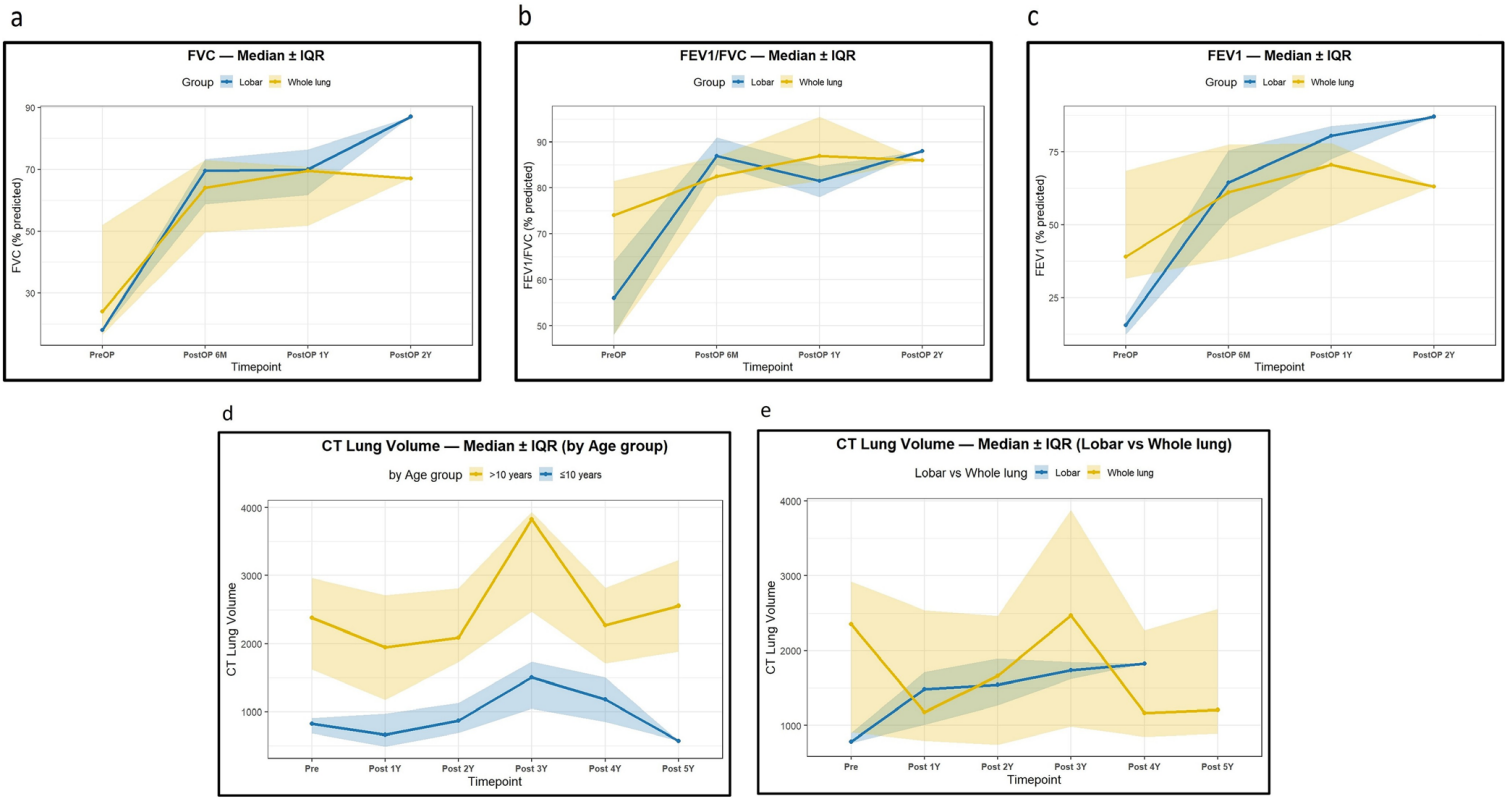
Longitudinal functional and volumetric adaptation after pediatric bilateral lung transplantation. (a–c) Pulmonary function changes stratified by graft type (lobar vs. whole‐lung transplantation). Both lobar and whole‐lung recipients showed progressive improvement in FVC (a), FEV_1_/FVC (b), and FEV_1_ (c) from pretransplant baseline to year 2. Although early variability differed modestly between graft types, the overall pattern of recovery was comparable across groups. (d) Lung volume by age group (≤ 10 vs. > 10 years). (e) Lung volume by graft type (lobar vs. whole‐lung). CT‐derived lung volumes (median ± IQR) increased after transplantation across all subgroups, with older and whole‐lung recipients exhibiting greater absolute volumes. CT, computed tomography; FEV_1_, forced expiratory volume in 1 s; FEV_1_/FVC, ratio of FEV_1_ to FVC; FVC, forced vital capacity; IQR, interquartile range; PostOP, postoperative; PreOP, preoperative; 6 M, 6 months; 1Y, 1 year; 2Y, 2 years.

Pulmonary function according to graft type is shown in Figure 2. Across all recipients, spirometry values improved substantially within 6 months of transplantation. Lobar and whole‐lung recipients also showed parallel improvements over time, with both groups exhibiting marked gains in FVC and FEV_1_. Overall, despite minor differences in the degree of change, patterns of pulmonary function recovery were broadly similar across graft type subgroups.

CT‐derived lung volume measurements are presented in Figure 2. Older recipients (> 10 years) had greater absolute lung volumes than younger recipients, reflecting expected differences in body size. Whole‐lung transplant recipients also demonstrated higher volumes than lobar transplant recipients at all time points. However, both age groups and graft types showed similar postoperative patterns, with gradual volume increases and peak expansion around 3 years. Overall, despite differences in absolute size, the temporal patterns of lung growth and remodeling were comparable across the subgroups.

### 3.5 Somatic Growth and Development Before and After Transplantation

**FIGURE 3:**
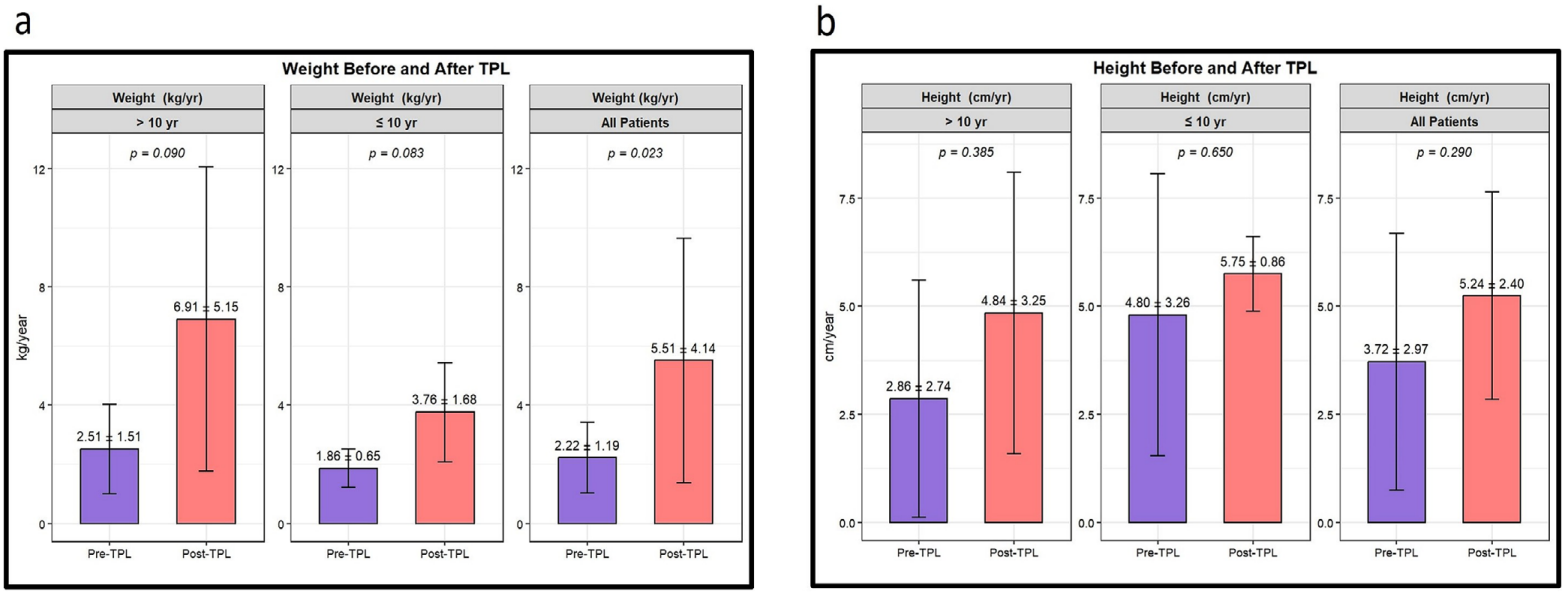
Somatic growth before and after pediatric lung transplantation. (a) Annualized weight velocity before and after transplantation. Weight gain increased after transplantation in both age groups, with several recipients demonstrating catch‐up growth following pretransplant growth retardation. (b) Annualized height velocity before and after transplantation. Height velocity improved after transplantation.TPL, transplantation.

Growth outcomes following transplantation are shown in Figure 3. Quantitative analyses of the annualized growth velocity showed that weight gain accelerated after transplantation in both age groups. The height velocity also improved after transplantation, although the difference was not statistically significant.

## 4 Discussion

In this single‐center cohort of pediatric recipients undergoing bilateral DDLT, we analyzed perioperative outcomes, longitudinal pulmonary function, CT‐derived volumetric adaptation, somatic growth patterns, and survival stratified by age and graft type. Overall, the patients demonstrated stable postoperative recovery across these domains, with the grafts demonstrating progressive structural and functional expansion over time. Notably, growth patterns that slowed before transplantation showed clear post‐transplant improvement, with many recipients returning to their individualized percentile curves.

The strengths of this study include its longitudinal evaluation of physiological, radiological, and developmental outcomes within a single institutional cohort, which enabled consistent perioperative management and follow‐up. Few previous investigations have examined these domains together across more than a decade of pediatric lung transplantation, particularly with subgroup comparisons by age and graft type. Building on this foundation, this study makes the following three key contributions to the field of pediatric lung transplantation: First, it offers new insights by demonstrating how functional recovery, graft remodeling, and growth trajectories evolve in parallel within the same cohort, thus clarifying the relationships that previous single‐domain studies could not address. Second, it provides a rare graft type comparison within DDLT, showing that lobar and whole‐lung transplantations yield comparable long‐term adaptations across multiple clinical domains. Third, it uniquely quantified the restoration of impaired pretransplant growth velocity, demonstrating a consistent catch‐up toward intrinsic growth patterns after transplantation.

Recent ISHLT Registry analyses have shown that pediatric lung transplant 1‐year survival in the modern era is consistently in the mid‐80% range, with long‐term mortality predominantly driven by CLAD [4, 12, 13]. Consistent with ISHLT registry reports, CLAD remained the major determinant of late mortality in our cohort, with survival curves beginning to decline approximately 4–5 years after transplantation [4, 13]. Importantly, previous studies evaluating the impact of age on pediatric lung transplantation have produced heterogeneous findings [14, 15]. Otani et al. [14] reported significantly poorer 1‐ and 5‐year OS in children aged 1–6 years than in those aged 7–17 years (70% vs. 100% at 1 year; 48% vs. 93% at 5 years). In contrast, other studies, including a 16‐year single‐center analysis of 117 recipients, demonstrated no significant differences in long‐term survival or CLAD‐free survival between children aged < 12 years and older recipients, despite higher perioperative complexity in the younger group (greater CPB and ECMO utilization) [15]. Our findings align more closely with these findings. Despite age‐related differences in baseline anthropometrics and perioperative course, the 5‐year OS and CLAD‐free survival did not differ significantly between younger and older recipients. This suggests that with modern perioperative support and multidisciplinary management, age alone may not represent a significant determinant of poor long‐term outcomes.

Pulmonary function improved substantially within 6 months of transplantation across all subgroups, regardless of graft type. Both lobar and whole‐lung recipients demonstrated broadly similar longitudinal improvements in FVC and FEV_1_. Although a slight, transient decline or a plateauing pattern was observed in several parameters after the first postoperative year, these chronological fluctuations did not represent meaningful functional deterioration and ultimately stabilized without significant clinical divergence between the two groups. This post‐year‐one stabilization is likely driven by an anthropometric mismatch during rapid pediatric growth spurts that temporarily outpaces allograft expansion, alongside the gradual resolution of early post‐transplant compensatory hyperinflation. Nonetheless, these findings reinforce the accumulating evidence that size‐reduced grafts, whether surgically downsized or transplanted as lobar grafts, can provide adequate and durable pulmonary function in children, mitigating the limitations imposed by donor–recipient size mismatch. Previous reports similarly demonstrated that pediatric recipients of size‐reduced or lobar grafts achieved meaningful postoperative lung function. Tanaka et al. showed that living‐donor lobar grafts exhibit postoperative improvement in vital capacity of nearly 60% and maintain long‐term functional performance, despite substantial initial size discrepancies between donor lobes and the pediatric thoracic cavity [5]. Similarly, Martens et al. [16] reported that volume‐reduced grafts in small children achieve acceptable early outcomes and demonstrate stable long‐term pulmonary function.

CT volumetry in our cohort demonstrated the expected differences in absolute lung volume between age groups and graft types; however, the temporal patterns of postoperative growth were broadly similar. Notably, the absolute lung volume exhibited a peak at approximately 3 years post‐transplant followed by a gradual, mild decline, which chronologically aligns with the clinical emergence of CLAD observed in our cohort. This subsequent volumetric contraction likely reflects early structural remodeling or fibroproliferative changes secondary to insidious CLAD development, rather than any intentional modifications in our institutional management. These observations extend prior limited reports, suggesting the potential for structural adaptation and compensatory expansion even in transplanted mature adult lobes. Tanaka et al. [5] similarly reported the progressive volumetric growth of transplanted adult lobes in children, indicating a preserved capacity for structural remodeling within the pediatric thorax. Recent reviews, including those by Ehrsam et al. [17], further highlight that transplanted lungs in children can undergo continued parenchymal expansion and functional maturation, which is far less pronounced in adults, reflecting the unique capacity of the pediatric thorax to promote compensatory graft growth. Collectively, our findings add to the emerging literature by demonstrating parallel functional and volumetric trajectories across graft types and age groups, further supporting the adaptability of transplanted lungs in growing children.

Consistent with prior literature describing growth retardation in chronically ill children awaiting transplantation, many recipients in our cohort exhibited plateauing or declining growth curves preoperatively [18, 19]. Following transplantation, both weight and height improved, with several children demonstrating a clear catch‐up toward baseline percentiles. This effect was particularly noticeable in older recipients, possibly because of their greater physiological reserve and shorter duration of preoperative growth impairment. Although patient height velocity increased postoperatively, the differences did not reach statistical significance, likely due to the small sample size and heterogeneous follow‐up intervals. These findings highlight the importance of incorporating structured nutritional and developmental monitoring in the long‐term management of pediatric transplant survivors.

This study had certain limitations. First, its retrospective design and small sample size limited statistical power, particularly for subgroup analyses, and follow‐up durations and measurement intervals were not fully uniform across participants. Consequently, our findings regarding functional, volumetric, and somatic growth trajectories should be interpreted as exploratory and descriptive rather than definitive proof of equivalence or superiority. Second, functional and volumetric assessments were constrained by age‐related testability in younger children, and spirometry and CT volumetry could not be standardized across the entire cohort. Third, the graft type and perioperative strategies were determined by clinical considerations rather than randomization, and the single‐center nature of the cohort may limit generalizability to other institutions.

In this 13‐year single‐center study, pediatric lung transplantation resulted in favorable functional recovery, appropriate graft adaptation, and improved somatic growth across age groups. Although younger recipients exhibited greater perioperative vulnerability, their long‐term and CLAD‐free survival rates were comparable to those of older children. While the outcomes between lobar and whole‐lung grafts appeared comparable within our cohort, these observations must be interpreted with caution given the small sample size, and further larger scale studies are essential to validate these survival and long‐term adaptation patterns.

## Author Contributions

Conceptualization: Samina Park and Seon Yong Bae; data curation: Seon Yong Bae; formal analysis: Seon Yong Bae; investigation: Seon Yong Bae; methodology: Samina Park and Seon Yong Bae; project administration: Samina Park; resources: Dae Hyeon Kim, Taeyoung Yun, Ji Hyeon Park, Bubse Na, Kwon Joong Na, Samina Park, Hyun Joo Lee, Ji Soo Park, Dong In Suh, Jimyung Park, In Kyu Park, Chang Hyun Kang, and Young Tae Kim; supervision: Kwon Joong Na, Samina Park, Hyun Joo Lee, Dong In Suh, In Kyu Park, Chang Hyun Kang, Young Tae Kim; validation: Samina Park and Seon Yong Bae; writing – original draft: Seon Yong Bae; writing – review and editing: Seon Yong Bae and Samina Park. Approval of final manuscript: All authors.

## Funding

The authors have nothing to report.

## Conflicts of Interest

The authors declare no conflicts of interest.

## Supporting information


**Table S1:** Preoperative and intraoperative characteristics of pediatric lung transplant recipients.
**Table S2:** Early postoperative outcomes and complications.

## Data Availability

The data that support the findings of this study are available on request from the corresponding author. The data are not publicly available due to privacy or ethical restrictions.
