# Supplementary material for: Functional, Volumetric, and Growth Outcomes After Pediatric Bilateral Lung Transplantation
Source: Pediatr Transplant. 2026 Aug 4;30(8):e70422. doi: 10.1111/petr.70422 (PMC13439153; doi:10.1111/petr.70422)
Supplement: Supplementary file 1 [file PETR-30-e70422-s001.docx]

**Supplementary Table**

**(Supplementary) E-Table 1. Preoperative and Intraoperative Characteristics of Pediatric Lung Transplant Recipients**

| **Variable** | **≤ 10 years (n = 7)** | **> 10 years (n = 9)** |
| --- | --- | --- |
| **Preoperative Characteristics** |  |  |
| Duration of waiting, days | 37 [1–68] | 58 [31.5–186] |
| Tracheostomy | 0 (0.0%) | 4 (44.4%) |
| Preoperative ECMO | 3 (42.9%) | 4 (44.4%) |
| **Type of pre/perioperative ECMO** |  |  |
| VA ECMO | 1 (33.3%) | 3 (75.0%) |
| VV ECMO | 1 (33.3%) | – |
| VAV ECMO | 1 (33.3%) | 1 (25.0%) |
| Duration of ECMO, days | 28.5 [1.25–58.75] | 38.5 [9.75–70.25] |
| **Intraoperative Characteristics** |  |  |
| **Incision type** |  |  |
| Clamshell | 6 (85.7%) | 9 (100%) |
| Anterior thoracotomy | 1 (14.3%) | 0 (0.0%) |
| **Intraoperative support** |  |  |
| CPB use | 2 (28.6%) | 3 (33.3%) |
| ECMO use | 5 (71.4%) | 6 (66.7%) |
| **Type of ECMO/CPB support** |  |  |
| Central VA ECMO | 3 (42.9%) | 8 (88.9%) |
| VAV ECMO | 1 (14.3%) | 1 (11.1%) |
| Peri VA ECMO | 2 (28.6%) | – |
| Avalon cannula | 1 (14.3%) | – |
| **Operative Parameters** |  |  |
| Right CIT, min | 150 [120–242] | 165 [118–187.5] |
| Right WIT, min | 63 [55–81] | 71 [46–119] |
| Left CIT, min | 295 [244–350] | 294 [215–312.5] |
| Left WIT, min | 60 [52–73] | 70 [49.5–78] |
| Operative time, min | 417 [365–550] | 465 [395–610] |

Values are presented as median [interquartile range] or number (percentage). *CPB, cardiopulmonary bypass; CIT, cold ischemic time; ECMO, extracorporeal membrane oxygenation; PAH, pulmonary arterial hypertension; VA, venoarterial; VAV, venoarterial–venous; VV, venovenous; and WIT, warm ischemic time*

**(Supplementary) E-Table 2. Early Postoperative Outcomes and Complications**

| **Variable** | **≤ 10 years (n = 7)** | **> 10 years (n = 9)** |
| --- | --- | --- |
| **Postoperative Support** |  |  |
| Postoperative ECMO | 3 (42.9%) | 4 (44.4%) |
| **Type of ECMO** |  |  |
| VV ECMO | 3 (100%) | 3 (75.0%) |
| Peri VA ECMO | – | 1 (25.0%) |
| **Duration of ECMO, days** | 12 [3–19] | 4.5 [2.5–8.0] |
| **Respiratory and ICU Course** |  |  |
| Duration of intubation, days | 9 [4–21] | 13 [4.5–18.5] |
| Duration of ICU stay, days | 9 [8–44] | 21 [13.5–30] |
| **Hospital stay, days** | 43.5 [27.8–88.5] | 62 [39.25–190.5] |
| **Tracheostomy after transplant** | 2 (28.6%) | 2 (22.2%) |
| **Postoperative Complications** |  |  |
| Reoperation for bleeding | 1 (14.3%) | 1 (11.1%) |
| 30-day mortality | 1 (14.3%) | 0 (0%) |
| Renal complication | 2 (28.6%) | 4 (44.4%) |
| Duration of CRRT, days | 27 | 14.5 [11.3–45.5] |
| Liver complication | 1 (14.3%) | 1 (11.1%) |
| Neurogenic complication | 5 (71.4%) | 2 (22.2%) |
| Retinopathy | 2 (28.6%) | 2 (22.2%) |
| Diabetes mellitus | 0 (0.0%) | 2 (22.2%) |

Values are presented as median [interquartile range] or number (percentage). *CRRT, continuous renal replacement therapy; VA, venoarterial; VV, venovenous; ICU, intensive care unit; ECMO, extracorporeal membrane oxygenation*
